# Characterization of the *Kingella kingae* Polysaccharide Capsule and Exopolysaccharide

**DOI:** 10.1371/journal.pone.0075409

**Published:** 2013-09-30

**Authors:** Kimberly F. Starr, Eric A. Porsch, Christian Heiss, Ian Black, Parastoo Azadi, Joseph W. St. Geme

**Affiliations:** 1 Department of Pediatrics and Department of Molecular Genetics and Microbiology, Duke University Medical Center, Durham, North Carolina, United States of America; 2 Complex Carbohydrate Research Center, University of Georgia, Athens, Georgia, United States of America; Monash University, Australia

## Abstract

Recent evidence indicates that *Kingella kingae* produces a polysaccharide capsule. In an effort to determine the composition and structure of this polysaccharide capsule, in the current study we purified capsular material from the surface of *K. kingae* strain 269–492 variant KK01 using acidic conditions to release the capsule and a series of steps to remove DNA, RNA, and protein. Analysis of the resulting material by gas chromatography and mass spectrometry revealed N-acetyl galactosamine (GalNAc), 3-deoxy-D-manno-oct-2-ulosonic acid (Kdo), and galactose (Gal). Further analysis by NMR demonstrated two distinct polysaccharides, one consisting of GalNAc and Kdo with the structure →3)-β-Gal*p*NAc-(1→5)-β-Kdo*p*-(2→ and the other containing galactose alone with the structure →5)-β-Gal*f*-(1→. Disruption of the *ctrA* gene required for surface localization of the *K. kingae* polysaccharide capsule resulted in elimination of GalNAc and Kdo but had no effect on the presence of Gal in bacterial surface extracts. In contrast, deletion of the *pamABCDE* locus involved in production of a reported galactan exopolysaccharide eliminated Gal but had no effect on the presence of GalNAc and Kdo in surface extracts. Disruption of *ctrA* and deletion of *pamABCDE* resulted in a loss of all carbohydrates in surface extracts. These results establish that *K. kingae* strain KK01 produces a polysaccharide capsule with the structure →3)-β-Gal*p*NAc-(1→5)-β-Kdo*p*-(2→ and a separate exopolysaccharide with the structure →5)-β-Gal*f*-(1→. The polysaccharide capsule and the exopolysaccharide require distinct genetic loci for surface localization.

## Introduction

*Kingella kingae* is a gram-negative bacterium that has emerged as a common cause of septic arthritis, osteomyelitis, and bacteremia in children 6–36 months of age [1]. Studies using culture-based and molecular-based detection strategies have established that *K. kingae* is a common commensal in the upper respiratory tract in young children [1]–[4]. In one report, *K. kingae* colonization of the pharynx was found in approximately 70% of children at some point during the first two years of life [5]. The pathogenesis of *K. kingae* disease is believed to involve bacterial translocation across the pharyngeal epithelial barrier, entry into the bloodstream, and dissemination to the joints, bones, or endocardium [4]. This pathogenic model is supported by data demonstrating genotypically identical isolates of *K. kingae* from the upper respiratory tract and either the bloodstream or joint fluid from patients with invasive disease [6], [7].

Extracellular polysaccharides are produced by many pathogenic bacteria and function as important virulence factors. These polysaccharides can be categorized generally as capsular polysaccharides or exopolysaccharides. Capsular polysaccharides are lipid-anchored outer membrane-associated carbohydrates and are involved in protection from host immune mechanisms, including phagocytosis and complement-mediated killing [8], [9]. These polysaccharides often exhibit a large degree of variation within a single bacterial species, with examples including *Escherichia coli* and *Streptococcus pneumoniae* [10]–[12]. In select cases, a specific capsule type can be associated with increased virulence potential, as highlighted by *Haemophilus influenzae* type b [13]. Exopolysaccharides are secreted carbohydrate polymers that are not anchored to the bacterial surface and that play a variety of roles, including modulation of adherence and modulation of biofilm formation [14], [15].

In recent work, we identified a polysaccharide capsule in *K. kingae* strain 269–492 that influences adhesive interactions with host cells and is dependent upon the *ctrABCD* ABC-type capsule export operon for surface localization [16]. In the current study, we set out to characterize the composition and structure of this polysaccharide capsule. Glycosyl analysis of bacterial surface extracts by gas chromatography/mass spectrometry and NMR revealed two distinct extracellular polysaccharides, including the polysaccharide capsule and a separate exopolysaccharide. The polysaccharide capsule contains N-acetyl galactosamine (GalNAc) and 3-deoxy-D-manno-oct-2-ulosonic acid (Kdo) and has the structure 3→)-β-Gal*p*NAc-(1→5)-β-Kdo*p*-(2→. In contrast, the exopolysaccharide contains only galactose and has the structure →5)-β-Gal*f*-(1→. Targeted mutagenesis established that the capsular polysaccharide and the exopolysaccharide require separate genetic loci for surface localization.

## Materials and Methods

### Bacterial Strains

The strains used in this study are listed in Table 1. *K. kingae* strain 269–492 was originally recovered from the joint fluid of a child with septic arthritis at St. Louis Children’s Hospital, St. Louis, MO. *K. kingae* KK01 is a stable sparsely piliated natural variant of strain 269–492 that forms nonspreading, non-corroding colonies and was used in all experiments in this study because of its colony morphology [17]. *K. kingae* strains were grown on chocolate agar plates at 37°C with 5% CO_2_ supplemented with 50 µg/ml kanamycin or 2 µg/ml erythromycin as appropriate. *K. kingae* strains were stored at −80°C in brain heart infusion with 30% glycerol. *E. coli* strain DH5α was used for construction of gene disruption plasmids. *E. coli* strains were routinely grown at 37°C on Luria-Bertani (LB) agar or in LB broth with 100 µg/ml ampicillin, 50 µg/ml kanamycin, or 500 µg/ml erythromycin as appropriate. *E. coli* strains were stored at −80°C in LB broth with 15% glycerol.

**Table 1:** Strains used in this study ?.

Strain	Description	Workcited
*K. kingae* strains		
269–492 (KK01)	Clinical isolate Spontaneous nonspreading, non-corroding colony variant of 269–492	[16]
KK01*pamABCDE.*	Deletion of *pamABCDE*	This work
KK01*ctrA*	Polar insertional mutation resulting in disruption of *ctrABCD* operon	[16]
KK01*ctrApamABCDE.*	Polar insertional mutation in *ctrA* and deletion of *pamABCDE*	This work
*E. coli* strains		
DH5α	*E. coli* F^−^ ?80d*lacZ*Δ*M15* Δ(*lacZYA-argF*)*U169 deoR recA1 endA1 hsdR17*(r_K_^−^ m_K_^+^) *phoA supE441 thi-1 gyrA96 relA1*	[32]

### Strain Construction

Targeted gene disruptions in *K. kingae* were generated as previously described [16], [18]. Briefly, plasmid-based gene disruption constructs were created in *E. coli*, linearized, and introduced into *K. kingae* strain KK01 via natural transformation. Transformants were recovered by plating on chocolate agar plates containing the appropriate antibiotic. Correct localization of gene disruptions was confirmed by PCR. The *ctrA* disruption was generated as described previously [16] but with the *aphA3* kanamycin resistance cassette as the marker. This disruption had a polar effect on the downstream genes in the *ctrABCD* operon. To delete the *pamABCDE* locus, fragments corresponding to the surrounding 5′ and 3′ regions of the locus were PCR amplified using the primers pam 5′for (GCGAATTCGGCGTTGGTGGAATATCCTG), pam 5′rev (GCGGATCCACCTTCTGGTCGCTGAAATG), pam 3′for (GCGGATCCTCAAAGGCTGGTATAAACAC), and pam 3′rev (GCAAGCTTCCATATCGCTTTGGCTTTGC), respectively. The resulting fragments were ligated into pUC19, creating puC19*pam* 5′+3′::BamHI. The *ermC* erythromycin cassette from pIDN4 was PCR amplified with flanking BamHI sites and ligated into pUC19*pam* 5′+3′, generating pUC19*pam*::*ermC*.

### Polysaccharide Extraction and Purification

For small scale capsule extractions, bacteria were washed and resuspended in either Tris acetate pH 5 (acid treatment for one hour) or PBS (heat treatment at 55°C for 1 hour). Cells were removed by centrifugation, and extracts were treated with proteinase K for one hour, and then concentrated as previously described [16].

To prepare strains for extraction of extracellular polysaccharide for purification, lawns of bacteria were grown overnight on chocolate agar plates. Subsequently, bacterial growth was swabbed from plates and resuspended in 50 ml of 1% formaldehyde in PBS to fix cells and allowed to stand for 15 minutes at room temperature. Bacteria were centrifuged at 4,355×g for 10 minutes and then resuspended in 40 ml of 50 mM Tris acetate pH 5. Following vigorous shaking for 1 hour at room temperature, bacteria were pelleted by centrifugation at 12,096×g for 20 minutes. The supernatant was recovered and was then filtered using a 0.22 µm filter. The filtered material was adjusted to pH 7 with 1 M Tris pH 9. To remove contaminating DNA, RNA, and protein, the filtered material was treated with 10 units of DNase (Fermentas) and 0.1 mg of RNase (Fermentas) at 37°C for 5 hours and then with 0.18 mg of proteinase K (Roche) at 55°C overnight. Samples were concentrated to 500 µl using 100 kDa MWCO filters and extracted once with Tris-saturated phenol pH 7.4 and twice with 100% chloroform. Extracted material was dialyzed extensively overnight in deionized water, flash frozen, and lyophilized. To purify exopolysaccharide galactan, we resuspended bacteria in PBS without formaldehyde, shook for one hour, pelleted the bacteria, and subjected the supernatant to the purification described above.

### Staining of Polysaccharide

Aliquots of the purified polysaccharide from *K. kingae* derivatives were separated on 10% SDS-PAGE gels. For Alcian blue staining, gels were stained with 0.125% Alcian blue as previously described [16]. For silver staining, gels were treated as previously described [19].

### Chemical Analysis

Monosaccharide composition analysis was performed by methanolysis and trimethylsilyl derivatization as previously described [20].

### NMR Spectroscopy

The isolated polysaccharide was deuterium-exchanged by lyophilization from D_2_O (99.9%D, Aldrich), dissolved in 270 µl D_2_O (99.96%D, Cambridge Isotope) containing 0.3 µl acetone, and placed in a 5-mm NMR tube with D_2_O-matched magnetic susceptibility plugs (Shigemi Inc.). One-dimensional proton and 2-D TOCSY and NOESY NMR spectra with solvent presaturation and gradient-enhanced COSY, HSQC, and HMBC NMR spectra were acquired on a Varian Inova 600 spectrometer at 50°C, equipped with a cryogenic triple-resonance probe. Spectral width was 2841 Hz in the proton and 18096 Hz in the carbon dimension. Mixing times were 150 ms for TOCSY and 300 ms for NOESY. The number of increments and scans, respectively were 512 and 4 for COSY, 200 and 8 for TOCSY, 200 and 16 for NOESY, 128 and 64 for HSQC, and 200 and 88 for HMBC. Chemical shifts were measured relative to acetone (δ_H_ = 2.218 ppm, δ_C_ = 33.0 ppm) [21].

## Results

### Polysaccharide can be Extracted and Purified from the Surface of *K. kingae* Strain 269–492 Variant KK01

**Figure 1:**
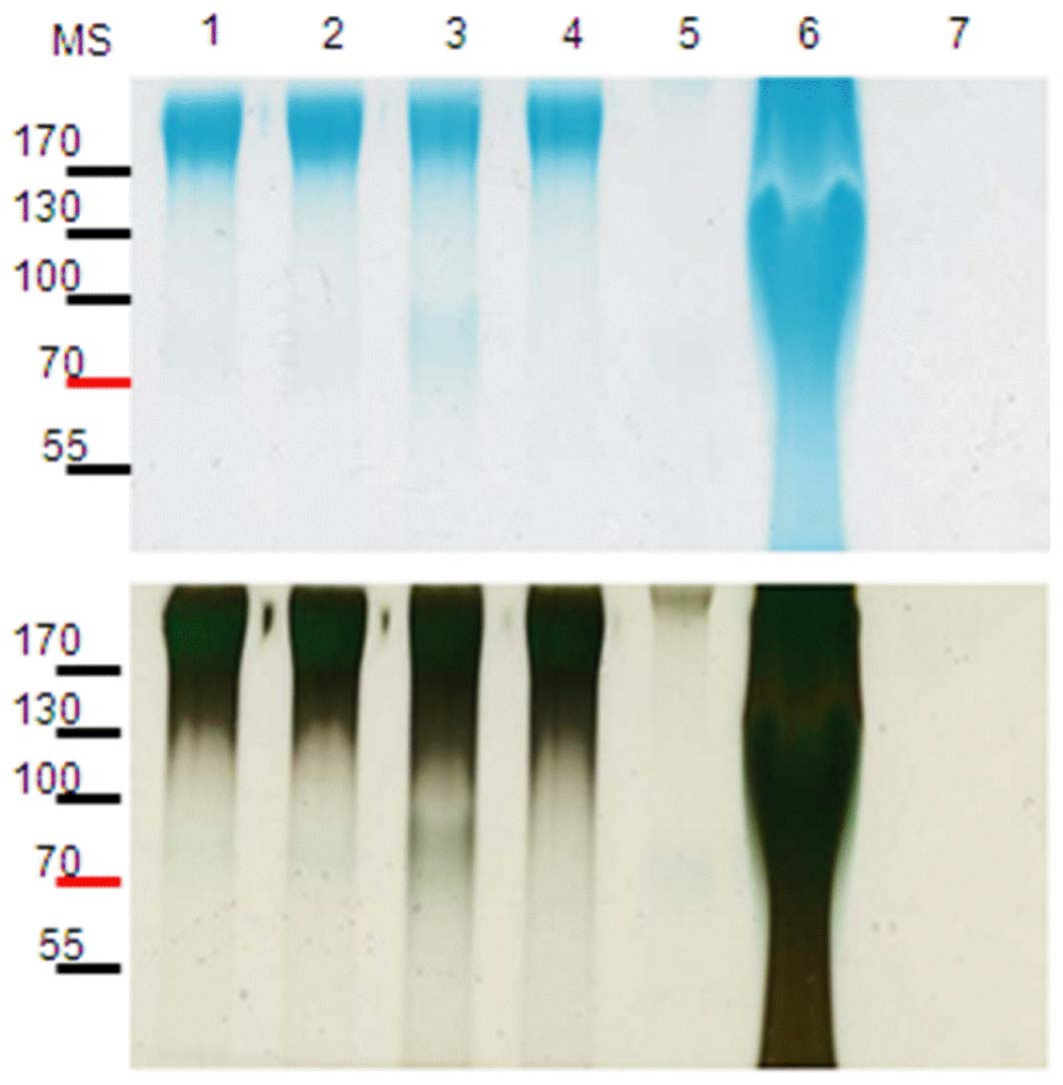
Staining profile and purity of polysaccharide material used for analysis. Alcian blue staining is shown on the top, and silver staining is on the bottom. Lane 1, extract from KK01 using heat; lane 2, extract from KK01 using Tris acetate; lane 3, purified material from KK01 using Tris acetate; lane 4, purified material from KK01*pamABCDE* using Tris acetate; lane 5, purified material from KK01*ctrA* using Tris acetate; lane 6, purified material from KK01*ctrA* using surface PBS extraction; lane 7, purified material from KK01*ctrA pamABCDE* using Tris acetate. MS = molecular size (in kDa).

In order to extract and purify the polysaccharide capsule, we incubated a bacterial suspension of *K. kingae* strain 269–492 variant KK01 in Tris acetate pH 5, aiming to dissociate the polysaccharide capsule from the bacterial surface by the acidic pH. To confirm that our extraction procedure yielded pure polysaccharide material, we examined the material by Alcian blue straining and silver staining (Figure 1). Alcian blue is a cationic dye and has been used previously to demonstrate the presence of the *K. kingae* capsule [16]. As shown in Figure 1, the extracted and purified material from the surface of *K. kingae* KK01 stained prominently with Alcian blue and silver reagents (lane 3), similar to control small scale heat and acid extracts from *K. kingae* KK01 (lanes 1–2). The Tris acetate extract from KK01*ctrA* (lacking a functional capsule export locus) failed to stain with either Alcian blue or silver reagents (lane 5), while the PBS surface extract from KK01*ctrA* stained well with Alcian blue and silver reagents (lane 6).

### *Kingella kingae* KK01 Extracellular Polysaccharide Contains GalNAc, Kdo, and Galactose

As shown in Table 2, composition analysis of the polysaccharide extracted from *K. kingae* KK01 revealed that the dominant components were GalNAc, Kdo, and galactose in roughly equimolar quantities. In addition, there were small quantities of xylose, glucose, N-acetyl glucosamine, and heptose, all common contaminants in this kind of preparation.

**Table 2:** Glycosyl composition of extracellular preparations of *K. kingae* strains used in this study.

Glycosyl residue	KK01 Trisacetate prep	KK01*pamABCDE*Tris acetate prep	KK01*ctrA* Trisacetate prep	KK01*ctrA* surfacePBS extraction	KK01*ctrA pamABCDE*Tris acetate prep
Xylose	0.3 (4.6)	1.0 (14.5)	9.0 (91.1)	0.1 (1.6)	10 (100)
Galactose	2.3 (27.1)	–	0.9 (7.5)	6.2 (64.5)	–
Glucose	0.2 (1.9)	–	0.1 (0.8)	1.8 (18.4)	–
N-Acetyl Galactosamine	3.0 (28.5)	4.2 (41.3)	–	0.1 (1.2)	–
N-Acetyl Glucosamine	0.4 (3.4)	–	–	0.4 (3.4)	–
Kdo*	3.4 (28.8)	4.8 (43.9)	–	0.8 (6.0)	–
Heptose	0.6 (5.6)	–	–	0.5 (4.8)	–

Mass in µg is listed first, and percentage is shown in parentheses. Mass and percentage are per 10 µg polysaccharide material analyzed.

* 3-Deoxy-D-Manno-oct-2-ulosonic acid.

**Figure 2:**
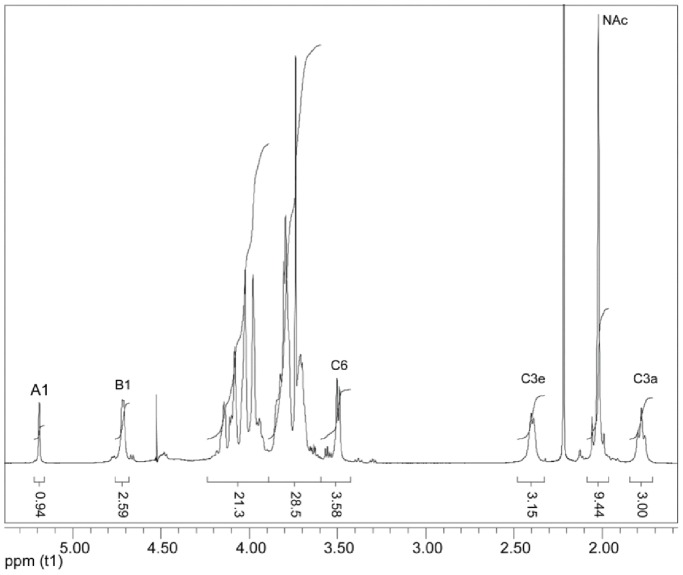
1-D proton NMR spectrum of the polysaccharide. The integration values show the relative molar ratio of the two polysaccharides, A_m_ and (B–C)_n_ of about 1∶3. For numbering, see Table 3 (C3e and C3a designate the equatorial and axial proton, respectively, of the C-3 methylene group).

**Figure 3:**
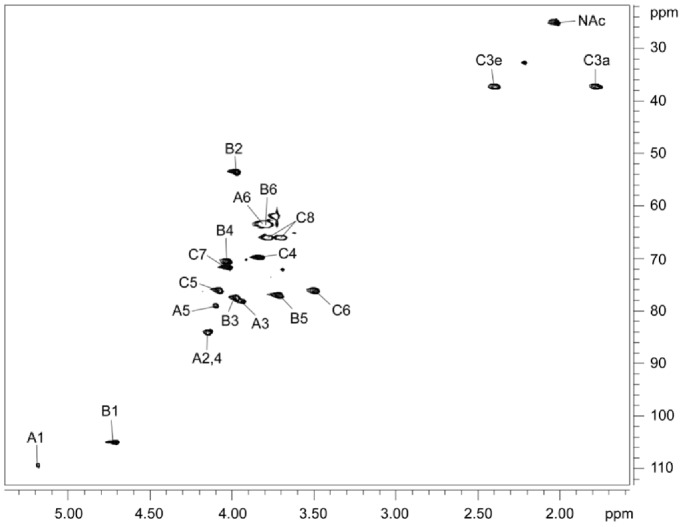
2-D HSQC NMR spectrum of the polysaccharide with assignments of all the signals of the three major residues. For numbering, see Table 3.

**Figure 4:**
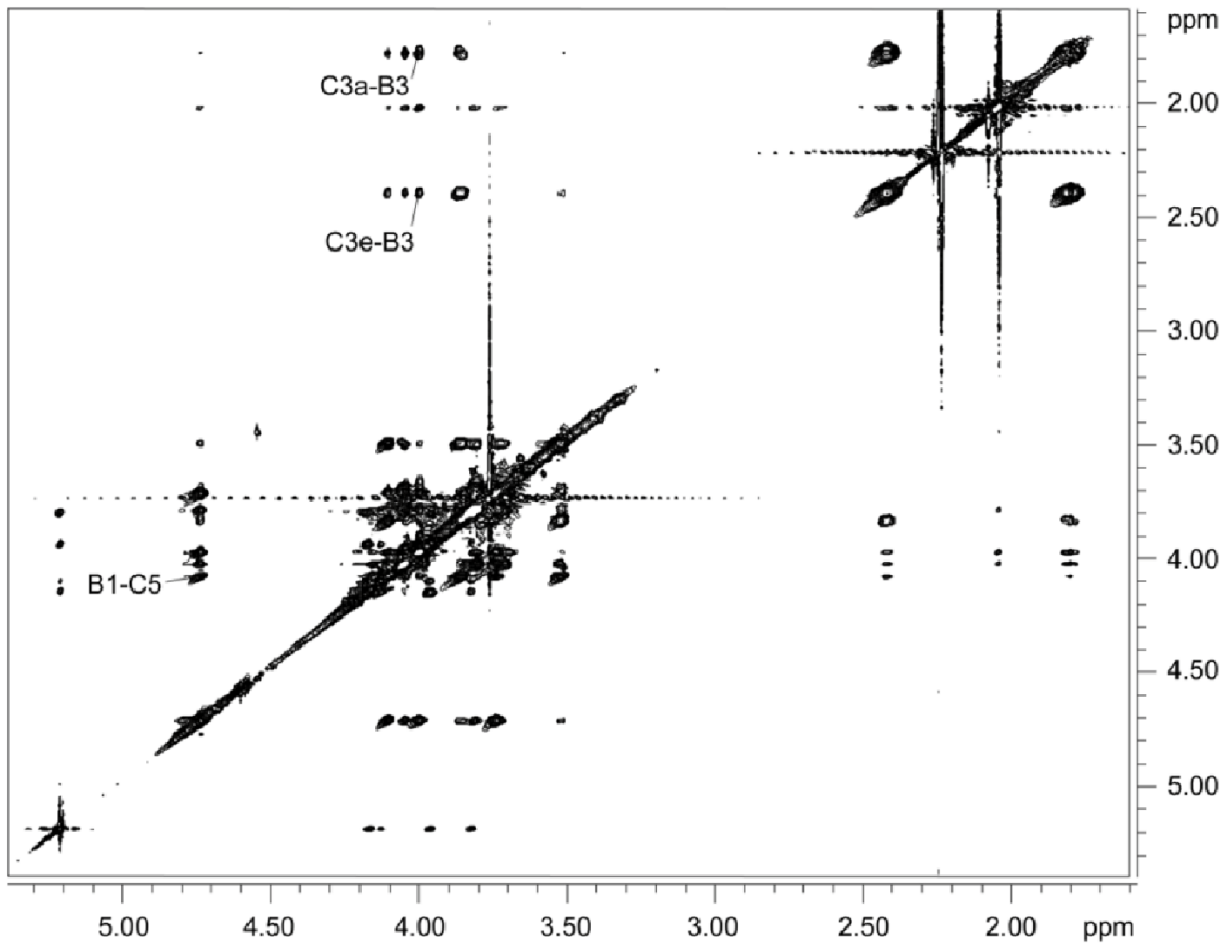
2-D NOESY NMR spectrum of the polysaccharide. The sequence-determining correlations are labeled. For numbering, see Table 3.

The 1-D proton NMR spectrum of the purified polysaccharide (Figure 2) showed two anomeric signals at 5.18 and 4.70 ppm, with an intensity ratio of 1∶3, and two upfield methylene signals at 2.40 and 1.77 ppm, each of equal intensity with the larger anomeric peak. The HSQC spectrum (Figure 3) showed that the signal at 5.18 ppm was associated with a carbon chemical shift of 109.4 ppm, implying that this residue was a furanose. Chemical shift assignment of the remaining nuclei (Table 3) demonstrated that the furanose had a galacto-configuration, and a 5.9-ppm downfield displacement of its C-5 chemical shift indicated that it was glycosylated in the 5-position. The further chemical shift assignments, based on 2-D COSY, TOCSY, and HSQC spectra, showed that the anomeric signal at 4.70 ppm belonged to a β-GalNAc residue and the methylene signals at 2.40 and 1.77 ppm belonged to a β-Kdo residue. Downfield shifts of the carbon nuclei involved in glycosidic linkages indicated that GalNAc was 3-linked and Kdo was 5-linked (see Table 3). Beside these major residues, small amounts (∼5%) of non-reducing end GalNAc and reducing end 5-α-Kdo were also detected and are likely the result of cleavage of some of the very acid labile Kdo glycosidic bonds during isolation. The NOESY spectrum (Figure 4) revealed NOE contacts between H-1 of GalNAc and H-5 of Kdo and between both H-3 s of Kdo and H-3 of GalNAc. The Gal*f* anomeric signal gave NOE contacts only to its own protons. HMBC showed a correlation between H-1 of GalNAc and C-5 of Kdo.

**Table 3:** Complete chemical shift assignment of the isolated polysaccharides.

No.	Residue	Chemical shift								NOE
		1	2	3	4	5	6	7	8	HMBC
A	5-β-Galf	5.18	4.15	3.94	4.15	4.1	3.82/3.82			
		**109.4**	84.1	78.5	84.1	**79.2**	62.1			A-5
B	3-β-GalpNAc	4.7	3.97	3.97	4.03	3.7	3.82/3.80			C-5
		**105.2**	53.6	**77.5**	70.5	77.1	63.5			C-5
C	5-β-Kdop	–	–	1.77/2.40	3.83	4.08	3.5	4.03	3.79/3.70	B-3
		174.3	**104.5**	37.2	70	**76.1**	76.1	71.9	65.8	

N-acetyl signal: 2.04/25.0 ppm.

Carbon chemical shifts are in italics. Carbon chemical shifts with characteristic downfield displacement due to glycosylation are in bold.

**Figure 5:**
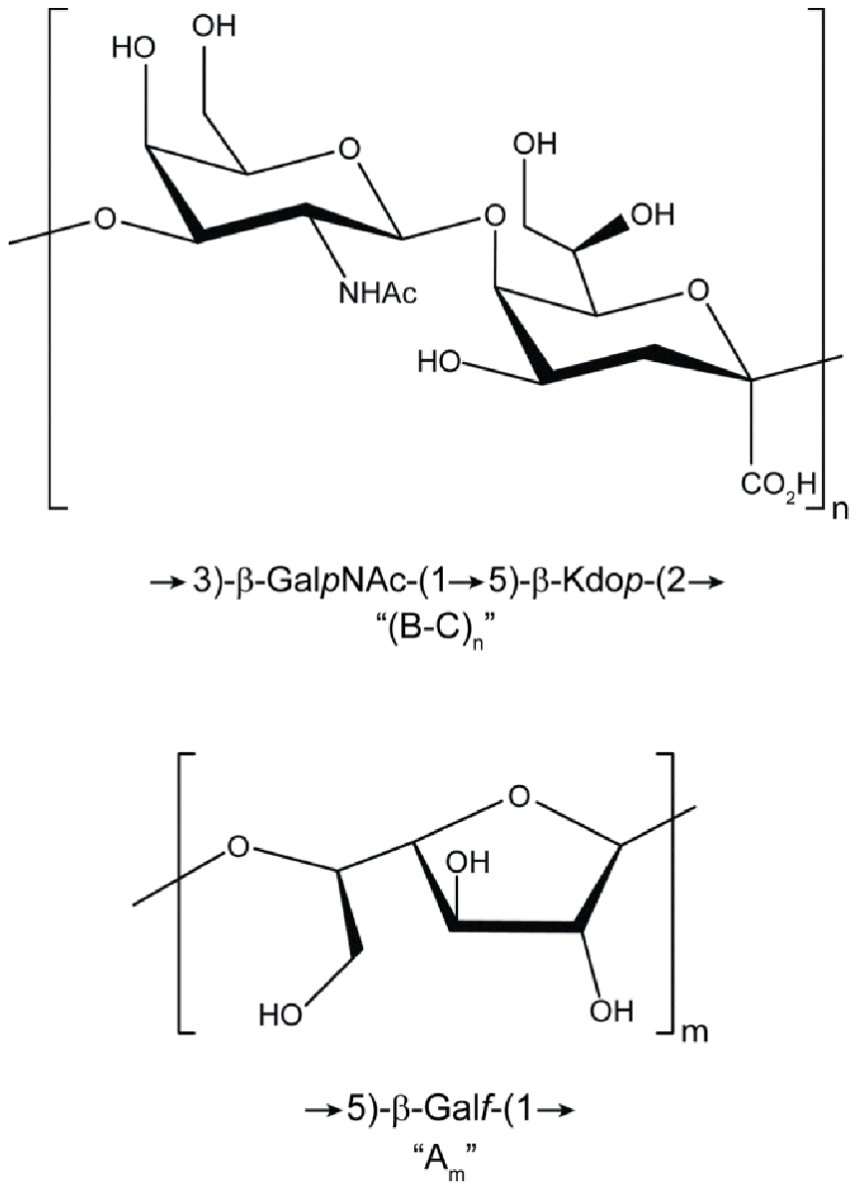
Structure of capsule polysaccharide repeating unit (top) and galactan exopolysaccharide repeating unit (bottom).

Taken together, these results demonstrated that the sample contained two polysaccharides, one composed of a disaccharide repeating unit with the structure →3)-β-Gal*p*NAc-(1→5)-β-Kdo*p*-(2→ and the other a galactofuranosyl homopolymer (galactan) with the structure →5)-β-Gal*f*-(1→ (Figure 5). The galactan homopolymer accounted for the galactose in the composition and methylation analyses and was present at about one-third the molar concentration of the GalNAc-Kdo polymer.

### The GalNAc-Kdo Polymer is the Polysaccharide Capsule and the Galactan Homopolymer is an Exopolysaccharide

**Figure 6:**
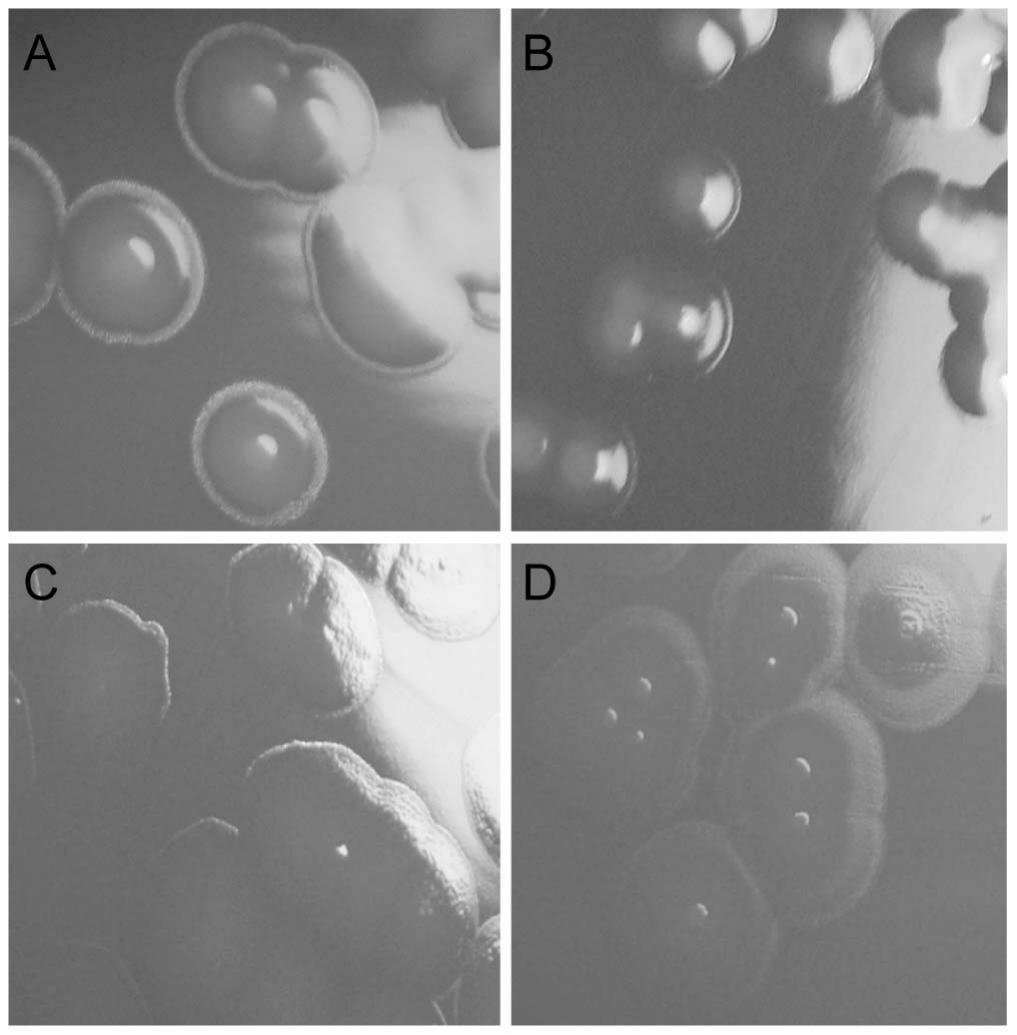
Colony morphology of *K. kingae* strains used in this study. Strain KK01 (A) and KK01*pamABCDE* (B) form mucoid colonies consistent with encapsulation. KK01*ctrA* (C) and KK01*ctrA pamABCDE* (D) form rough, non-mucoid colonies consistent with loss of encapsulation.

In a recent report, *K. kingae* strain PYKK181 was found to produce an exopolysaccharide that is a galactan homopolymer and is encoded by the *pamABCDE* locus [22]. To elucidate the relationship between the *K. kingae* polysaccharide capsule and the two distinct polysaccharide structures present in our bacterial surface extracts, we began by creating a derivative of KK01 with a deletion of *pamABCDE.* As shown in Figure 1 (lane 4) and Table 2, the polysaccharide extract from KK01*pamABCDE* stained with Alcian blue, contained large amounts of GalNAc and Kdo, and had only trace quantities of galactose, suggesting that the →5)-β-Gal*f*-(1→ homopolymer corresponds to the galactan exopolysaccharide present in *K. kingae* strain PYKK181. As shown in Figure 6, examination of the colony morphology of KK01*pamABCDE* revealed mucoid colonies, indicating encapsulation and suggesting that the GalNAc-Kdo polysaccharide corresponds to the polysaccharide capsule. To confirm these conclusions, we examined polysaccharide extracts from strain KK01*ctrA*, recognizing that the *ctrA* gene is required for export and surface localization of the polysaccharide capsule. Interestingly, glycosyl composition analysis revealed no GalNAc or Kdo and minimal amounts of galactose compared to the quantities of contaminating xylose and glucose (Table 2), raising the possibility that either the absence of the GalNAc-Kdo polysaccharide results in free release of the galactan exopolysaccharide or that *ctrA* is essential for surface localization of both the GalNAc-Kdo polysaccharide and the galactan homopolymer. To address these possibilities, we modified the extraction procedure to eliminate both the initial fixation step and the Tris acetate treatment, aiming to recover loosely associated polysaccharide from strain KK01*ctrA*. Using this technique, a large quantity of galactose was detected in the purified material and only minimal amounts of GalNAc and Kdo were present (probably reflecting some bacterial lysis) (Table 2), indicating that strain KK01*ctrA* localizes the →5)-β-Gal*f*-(1→ polymer but not the →3)-β-Gal*p*NAc-(1→5)-β-Kdo*p*-(2→ polymer on the bacterial surface. Consistent with our previous work, examination of the colony morphology of KK01*ctrA* revealed non-mucoid colonies (Figure 6), indicating lack of a capsule. Analysis of the KK01*ctrA pamABCDE* double mutant revealed no Alcian blue staining material, complete loss of GalNAc, Kdo, and galactose, and non-mucoid colonies (Figure 1, Table 2, and Figure 6). Together these results provide strong evidence that the GalNAc-Kdo polysaccharide is the polysaccharide capsule and that the galactan polymer is an exopolysaccharide.

## Discussion

In previous work we showed that *K. kingae* produces a polysaccharide capsule and contains a capsule export locus designated *ctrABCD* with high homology to the capsule export locus in *N. meningitidis*. In addition, we demonstrated that the *K. kingae* capsule is associated with a mucoid colony phenotype on chocolate agar and is visible by thin section transmission electron microscopy after staining with cationic ferritin [16]. In the present study we set out to characterize the composition and structure of the *K. kingae* polysaccharide capsule. As a first step, we developed a capsule extraction and purification procedure that generated material amenable to analysis by GC/MS and NMR. Examination of the extracted material from *K. kingae* strain KK01 revealed GalNAc, Kdo, and galactose in roughly equimolar amounts. Additional analysis of the extracted material from derivatives of strain KK01 containing targeted mutations in the capsule export locus, the galactan biosynthesis locus, or both loci established that GalNAc and Kdo make up the capsule and that galactose makes up a galactan exopolysaccharide.

In considering our results, it is noteworthy that Bendaoud et al. reported two polysaccharides in the supernatant from *K. kingae* strain PYKK181, including a polysaccharide with the structure →6)-α-D-Glc*p*NAc-(1→5)-β-Kdo*p*-(2→ and a galactan homopolymer with the structure →3)-β-Gal*f*-(1→6)-β-Gal*f*-(1→ that was designated *p*oly-DNA-containing *a*nti-adhesive *m*aterial extract (PAM galactan) and was found to inhibit biofilm formation by a variety of organisms [22]. In the report by Bendaoud and coworkers, the specific relationship between these two polysaccharides and the organism was unclear. Based on the results of our mutational studies, we believe that the GlcNAc-Kdo polymer from strain PYKK181 is a polysaccharide capsule and that the PAM galactan is an exopolysaccharide.

It is interesting that the strain KK01 polysaccharide capsule has the structure →3)-β-Gal*p*NAc-(1→5)-β-Kdop-(2→ and the strain PYKK181 polysaccharide capsule has the structure →6)-α-D-Glc*p*NAc-(1→5)-β-Kdo*p*-(2→ [22]. The fact that the polysaccharide capsules expressed by these strains have different structures raises the possibility that there may be multiple *K. kingae* capsule types, similar to the case with *E. coli, S. pneumoniae, N. meningitidis, H. influenzae,* and a number of other bacterial pathogens [10], [11], [23]. Along these lines, it is notable that Amit et al. observed an association between *K. kingae* PFGE clonal types and specific clinical presentations [24]. In ongoing work, we are examining whether there is variability in capsule type among different clones and conservation of capsule type within a given clone. It is possible that certain capsule types confer greater virulence than others and are associated with specific disease processes.

Both the strain KK01 polysaccharide capsule and the strain PYKK181 polysaccharide capsule contain Kdo, a molecule that is well recognized as an essential component of lipopolysaccharide and is also present in a small number of bacterial polysaccharide capsules. The capsule produced by strain KK01 has the same structure as the capsule produced by *Moraxella nonliquefaciens* strain 3828/60 [25] and has the same composition but different glycosyl linkages compared to the capsules produced by *E. coli* K14 [→6)-β-D-Gal*p*NAc-(1→5)-β-Kdo*p*(2→] [26] and *N. meningitidis* serogroup 29E [→3)-α-D-Gal*p*NAc-(1→7)-β-Kdo*p*(2→] [27]. Other examples of bacterial polysaccharide capsules that contain Kdo include the capsule produced by *Actinobacillus pleuropneumoniae* serotype 5a (identical to the capsule produced by *K. kingae* strain PYKK181) [28], the capsule produced by *Sinorhizobium meliloti* strain 1021 with the structure →7)-β-Kdo*p*(2→ [29], and the capsule expressed by *E. coli* K15 with the structure →4)-α-D-Glc*p*NAc-(1→5)-β-Kdo*p*(2→ [30].

In considering the genetic determinants of *K. kingae* polysaccharide capsule biosynthesis, it is notable that the *ctrABCD* capsule export locus is not flanked by a capsule synthesis operon, thus differing from the configuration of capsule genes in most other bacteria that produce a polysaccharide capsule [8], [9], [31]. Based on available *K. kingae* genome data, there is no apparent capsular polysaccharide synthesis operon. We speculate that the genes required for synthesis of the capsular polysaccharide are not arranged in an operon and instead are located at separate loci throughout the genome.

Sequence analysis of the PAM locus in *K. kingae* strain KK01 and the PAM locus in *K. kingae* strain PYKK 181 reveals almost complete identity. Despite this sequence conservation, the structures of the galactan homopolymers in these strains are different, with distinct linkages. One possibility is that there are other genes beyond *pamABCDE* involved in biosynthesis of the galactan exopolysaccharides. Alternatively, the limited sequence variation may result in significant changes in enzymatic specificity.

To summarize, in this study we have established that *K. kingae* expresses two extracellular polysaccharides that require distinct genetic loci for surface localization, including a polysaccharide capsule and an exopolysaccharide. In future studies we will examine the contribution of these polysaccharides to the pathogenesis of *K. kingae* disease.
